# A road map for neglected tropical diseases 2021–2030

**DOI:** 10.1093/trstmh/trab002

**Published:** 2021-01-28

**Authors:** Mwelecele N Malecela, Camilla Ducker

**Affiliations:** Department of Control of Neglected Tropical Diseases, World Health Organization, 1211 Geneva, Switzerland; Department of Control of Neglected Tropical Diseases, World Health Organization, 1211 Geneva, Switzerland

**Keywords:** neglected tropical diseases, road map, integration

To neglect something is to fail to take care of it properly, particularly through inattention. Twenty diseases and disease groups comprise the WHO's portfolio of neglected tropical diseases (NTDs) and each could provide a case study for that definition. Historical inattention by policymakers to these disabling, disfiguring and sometimes lethal health problems is probably explained by the fact that most NTDs predominantly affect people whose individual political power is wielded only very lightly, if at all.

The 20 NTDs are each aetiologically, epidemiologically and clinically unique. It can be difficult, at times, to gather them together. They do share one defining characteristic, however: they impact the poorest communities and trap those communities in cycles of poverty. They collectively affect more than 1 billion people in such communities worldwide, heaping devastating health, social and economic consequences on affected individuals and their families.

Since 2010, significant progress has been made, catalysed in part by a road map for intervention published by the WHO in 2012.^1^ Today, 500 million people no longer require interventions against one or more NTDs, and 42 countries, territories and areas have eliminated at least one NTD.^2^ Dracunculiasis is on the verge of eradication, with just 54 human cases reported in four countries in 2019. Lymphatic filariasis and trachoma have been eliminated as public health problems in 16 and 10 countries, respectively. Onchocerciasis has been eliminated in four countries. The annual number of cases of human African trypanosomiasis has fallen from more than 7000 in 2012 to fewer than 1000 in 2018, eclipsing the previous target of 2000 cases by 2020. The number of new leprosy cases reported globally has also continued to decline since 2010, at an average of 1% per year.

These are notable achievements. They have required some extraordinary efforts at all levels. They should in no way be underestimated or downplayed.

They should not obscure either, however, the fact that an immense task remains before us.

The road map for NTDs 2021–2030^2^ sets out global targets and milestones to prevent, control, eliminate and eradicate these diseases. It also defines cross-cutting targets, aligned with the WHO's 13th General Programme of Work (2019–2023) and the United Nations’ Sustainable Development Goals (SDGs).^3^ Crucially, the road map proposes strategies for attaining these targets over the next decade.^2^

This new road map was drafted after an extensive global consultation that began in 2018 and culminated in the document's endorsement by member states at the 73th World Health Assembly in November 2020. The process involved regional workshops with managers of NTD programmes, country workshops with stakeholders in NTDs and related areas of work, as well as detailed input from disease experts, disease modellers, donors and partners. More than 100 bilateral interviews were conducted and more than 300 written responses, from two rounds of online consultations, were received and analysed.

It is to be hoped, therefore, that the new road map is an accurate and thorough depiction of the perspectives of member states and a wide range of their supporters. The process was designed to ensure that the vision embodied in the 2021–2030 road map belongs not to the WHO alone, but to the entire global community dedicated to fighting NTDs.

As the NTD community began to plan and develop the 2021–2030 road map, it became clear that fundamental shifts in strategy were indicated. Three shifts, in fact, form its pillars. The first is a shift from measuring process to measuring impact. The second is a shift from vertical programming to horizontal, cross-cutting programming. The third shift, and potentially the most significant, calls for a move away from partner-led to country-driven and country-owned work.

During the road map's development, a thorough review of existing evidence was undertaken to identify and analyse critical gaps—both within and across diseases—which have led to bottlenecks in progress. Without addressing these, no meaningful advance seems likely. This resulted in the production of a heat map, offering an at-a-glance view of the global position.^2^ As the road map is rolled out, the WHO and partners will support countries to develop analogous national-level heat maps, allowing investments to be directed to areas where they will have the greatest possible impact. This may be particularly vital when it comes to the link between operational research and programmatic implementation.^4^ In this respect, innovation is, naturally, vitally important, but it must also be context-driven. One area highlighted by the heat map across multiple NTDs was diagnostics. This has led directly to the formation of a new WHO Diagnostic Technical Advisory Group for NTDs, which has already commenced work.^5^ Given the limited resources available in the field of diagnostic development, it is imperative that such activities are aligned with the road map and its targets.^6^

Of course, individual diagnostic assays are only one piece of the larger monitoring and evaluation puzzle. To estimate population-level incidence or prevalence, epidemiological rigour, consistency between settings, as well as quality assurance and quality control processes like those routinely applied by Tropical Data,^7^ are essential. The road map's companion document, ‘A monitoring and evaluation framework for neglected tropical diseases 2021–2030’, is intended to help programmes consider these and other issues. Monitoring of progress towards achievement of road map targets will be a continuous process over the next 10 y, building on and strengthening existing data processes at subnational, national, regional and global levels. Technical support to mainstream NTD data into stronger national health information systems, and powerful integrated platforms to streamline and facilitate data reporting and processing at regional and global levels, such as the WHO's Preventive Chemotherapy Joint Application Package, the WHO Integrated Data Platform or the future country portal of the WHO Health Data Hub, will continue to be provided. There will also be a periodic reassessment of gaps every 2 y, focused on the 11 dimensions defined in the heat map.^2^ A comprehensive review, including progress against the 2030 targets plus gap reassessment, will be carried out in 2022, 2024, 2026, 2029 and 2031, following World Health Assembly reporting requirements.^8^

The world has changed since 2012, when the first global road map on NTDs was published.^2^ It has changed even more since work began on developing the new one.^9^ It is conceivable that even donors who have in recent years staunchly supported work against NTDs will be re-examining their priorities, and that development aid in general will be scarce. Several of the road map's other companion pieces, namely, the investment case and sustainability framework, may help to unlock alternative sources of financial and non-financial resources. Evidence-based investment cases are an increasingly important tool for resource mobilisation both at global and national levels. For this reason, development of an investment case was undertaken hand-in-hand with development of the new road map. The investment case should appeal to both domestic ministries of health and finance and international developmental agencies, partners and non-governmental organisations, encouraging expanded investment in control and elimination of NTDs.

In addition to fitting squarely within the overarching framework of the SDGs, the 2021–2030 NTD road map and its targets fully align with the renewed emphasis on universal health coverage among the global public health community. The road map is built on the principle of impact at country level through cross-cutting approaches, owned and driven by countries themselves, and augmented by coordinated support from partners. The investment case will support this phased transition without compromising our collective ability to deliver the anticipated reductions in NTD incidence and prevalence. Financing priorities are defined across the 20 NTDs; the financing required to sustainably tackle all endemic NTDs is analysed with reference to a set of country-level case studies. The sustainability framework, for its part, seeks to ensure that countries can work practically towards embedding NTDs in their health systems, and that financial and non-financial contributions can be tracked with proper accountability. Mutual accountability between countries, partners and the WHO will promote true partnership en route to achieving the 2030 targets.

The shift to more collaborative and cross-sectoral approaches arises from necessity as well as principle. Uncertain times, along with the need to ensure programmatic acceleration, require us all to work more closely and collaboratively across sectors. To take one concrete example, water, sanitation and hygiene (WASH) play a key role in the struggle against NTDs and COVID-19 has brought to the fore the fundamental role that safe water, adequate sanitation and hygienic conditions play in preserving and protecting human health. And yet we know only too well that a great many of the communities we work with have grossly inadequate access to WASH. One of the road map's many ambitious targets is to ensure that 100% of all NTD-endemic communities have access to at least basic WASH services by 2030.

Other areas of collaboration will also be critical, including greater integration between NTDs and horizon-scanning towards the effects of climate change on NTD-affected populations. The new road map envisages a systematic, interdisciplinary and cross-sectoral strategy to prevent, control and eliminate NTDs, employing a One Health approach and the measures articulated in the Global Vector Control Response 2017–2030.

In all the talk, however, of collaboration and accountability, we should never forget that our most important collaborators are the individuals who are or might be affected by NTDs, and that our ultimate accountability is to them. Millions of lives will be improved, prolonged and saved if and when our work comes to fruition, or diminished and lost if we fail. Our targets for eradicating, eliminating or controlling NTDs are now set, and we must agree that it is unacceptable for individuals, families and communities to bear their awful burden, or to be inadvertently harmed by suboptimal interventions.

That is why we set such store by working together and continuing to innovate, always with a focus on the human lives behind the data. This requires investment in NTDs, of course—by endemic countries, in their workforce and infrastructure—and by donors, non-governmental organisations and technical partners. It is in all our interests, however, to ensure long-term investment in the foundations needed for equitable, accessible and rights-based primary healthcare.

We believe in our 2021–2030 NTD road map. It represents a shared vision and pushes for timely shifts in the NTD community's core operating model. Its targets are necessarily ambitious. Reaching all those in need is now our daunting task but we cannot and will not shirk our responsibility.

## Data Availability

None.
